# IoT Traffic Analyzer Tool with Automated and Holistic Feature Extraction Capability

**DOI:** 10.3390/s23115011

**Published:** 2023-05-23

**Authors:** Alanoud Subahi, Miada Almasre

**Affiliations:** 1 Faculty of Computing and Information Technology, Department of Information Technology, King Abdulaziz University, Rabigh 25732, Saudi Arabia; 2 Faculty of Computing and Information Technology, Department of Information Technology, King Abdulaziz University, Jeddah 21589, Saudi Arabia

**Keywords:** IoT network traffic, IoT traffic analysis, IoT automatic feature extraction, holistic traffic analysis

## Abstract

The Internet of Things (IoT) is an emerging technology that attracted considerable attention in the last decade to become one of the most researched topics in computer science studies. This research aims to develop a benchmark framework for a public multi-task IoT traffic analyzer tool that holistically extracts network traffic features from an IoT device in a smart home environment that researchers in various IoT industries can implement to collect information about IoT network behavior. A custom testbed with four IoT devices is created to collect real-time network traffic data based on seventeen comprehensive scenarios of these devices’ possible interactions. The output data is fed into the IoT traffic analyzer tool for both flow and packet levels analysis to extract all possible features. Such features are ultimately classified into five categories: IoT device type, IoT device behavior, Human interaction type, IoT behavior within the network, and Abnormal behavior. The tool is then evaluated by 20 users considering three variables: usefulness, accuracy of information being extracted, performance and usability. Users in three groups were highly satisfied with the interface and ease of use of the tool, with scores ranging from 90.5% to 93.8% and with an average score between 4.52 and 4.69 with a low standard deviation range, indicating that most of the data revolve around the mean

## 1. Introduction

From smart homes to smart cities, IoT has become a distinct facet of an emerging and continuously evolving ecosystem that redefines traditional paradigms of cyber-systems that requires computing devices as mediums for networking and information sharing. In an IoT ecosystem, non-computing devices are enhanced with IoT nodes (e.g., sensors) that permit a high-level, inherently distributed form of connectivity over the Internet. Applications of IoT paradigms of connectivity are currently being utilized in such domains [1] as homes, industry, cities, medicine, transportation, and buildings, i.e., smart energy management [2], and building controls [3]. Recently, many objects around us are linked to the networks under the IoT paradigm to intelligently identify, locate, track, monitor, and manage things, such as healthcare, occupancy, and transportation [4,5,6]. Each of these smart devices is capable of seamless communication through sensors to share and transfer information over a network with minimal or no requiring human intervention [7].

Understandably, IoT application domains use various wireless communication protocols as they communicate over the Internet [8]. Subsequently, IoT devices generate data flows that users rarely monitor. They collect and store a vast amount of data about individuals and organizations, exposing end-users to all cyber threats. Such threats are not limited only to attacks on privacy; they can also extend to attacks on network availability and performance. Investigating the performance or the behavior of IoT devices depends firstly on analyzing their traffic and secondly on extracting all of the features that might be useful in characterizing their information flow. While there has been extensive research into characterizing general Internet traffic for security and privacy purposes, few studies have focused specifically on IoT traffic [9]. It is important to note that network features of non-IoT traffic cannot be relied upon to predict the behavior of IoT traffic, as these two types of traffic differ significantly, an aspect that researchers rarely observe. According to [10,11], it is important to note that IoT network traffic varies from non-IoT traffic, such as those from smartphones or PCs. This is due to the distinct features and behavior of IoT devices, which are not expected to perform like PCs. Researchers, i.e., Refs. [12,13] have identified eight network features that distinguish between the behavior of IoT devices and non-IoT devices, such as smartphones or PCs.

Our research delves deeply into the use of IoT network features for various purposes. We have observed some important issues. Firstly, although some current IoT research may share almost similar contributions, such as identifying the type and behavior of an IoT device [14] or understanding its fingerprint [15], methods used in analyzing and extracting features from IoT traffic differ, depending on the specific goal of the research. Secondly, we have found that not all IoT researchers have the same level of knowledge about IoT network features, such as their significance, usage, purpose, or weight, which can have a negative impact on their research outcomes. Despite this, researchers still spend significant time and effort extracting IoT network features and understanding their importance in order to identify features that suit their research. Finally, we have noticed that many IoT researchers tend to focus on particular features rather than taking a comprehensive approach to identifying the usage and importance of each feature [15,16].

From the observations made, our research emphasizes the importance of researchers having a better understanding and awareness of IoT network features to achieve more effective and impactful research outcomes. Therefore, the development of a tool capable of extracting all IoT device features is a major research focus. This will lead to more precise judgments on IoT traffic in a shorter time, promoting best practices in research within this field.

Toward this end, this research proposes a new public IoT traffic analyzer tool that aims to enhance security in smart homes. This tool offers a comprehensive approach to extracting all possible traffic features. It is designed to benefit the IoT research community by providing them with a useful tool that can speed up their research time and help them focus on their contributions. Unlike other IoT research that targets consumers, the beneficiaries of this research are the IoT researchers themselves. The tool generates a list of extracted IoT network features, making it easier for researchers to select only the relevant features for their research. This innovative tool is expected to assist and support IoT researchers in achieving their goals efficiently. The contributions of this paper can be summarized as follows:

- Proposed a real-time and comprehensive network traffic generation framework for gathering and analyzing four IoT devices in a smart home environment. Such framework is comprehensive in the sense that it covers all different scenarios of collecting the IoT traffic that goes from the IoT device to its cloud server or/and from the IoT device to other IoT device/s within the same domain and vice versa.
- Built an innovative multi-task IoT traffic analyzer tool that holistically analyzes pre-collected IoT network traffic both on flow and pact levels and extracts all possible IoT network features, including statistical and header feature sets from different network layers.
- Classified and group the extracted features into three different CSV files, then explain each feature’s importance, usage, and usefulness. For example, features that identify: the behavior or profiling of the IoT device, or if there is any interaction between the human and the IoT device, between the IoT device and its cloud, or between the IoT device and other IoT device/s, or identify the attack as well as the attack type.

The rest of the paper is organized as follows: Section 2 highlights recent research in IoT traffic analysis. In Section 3, we discussed how to collect smart-home traffic in our proposed model, followed by a detailed description of the method we use to build the tool. Section 4. Presents the results, discussion, and evaluation of implementing the tool. Finally, the Conclusion and Future work is discussed in Section 5.

## 2. Literature Review

In this research, we conduct a deep study to cover almost all IoT research with different contributions. This section presents recent IoT research that applied different methods to collect and analyze IoT traffic and extract its features for many purposes. Section 2.1 examines the studies that applied several feature extraction methodologies and the main objectives of extracting such features, while Section 2.2 examines the studies that used various forms of creating IoT datasets for several contributions.

### 2.1. Extracting Features Methodology

To the best of our knowledge, almost all IoT researches are focused on either recognizing IoT devices from non-IoT devices, detecting abnormal behaviors of the IoT traffic, classifying the type of IoT device, creating a unique fingerprint and profile for each IoT device, creating Intrusion Detection System to protect the IoT system, or creating a real-time risk assessment system for the IoT device. Table 1 summarizes the contribution of some IoT papers, the methods used to achieve such contributions, as well as the extracted features.

For example, the main objective of extracting IoT features in [15] is to identify the type and model of IoT devices. The authors profiled the devices based on the communication pattern of each device by adopting the header information extracted from their network packets. They calculated the header information’s similarity using Euclidean distance-based metric. They claim that their method is feasible because each IoT device has a distinct communication pattern.

While in [16], the authors discussed existing approaches for behavioral fingerprinting. Their objectives were to extract IoT features to help researchers understand the behavior of the IoT device and establish several guidelines related to the device operations.

On the one hand, the authors of [14] adopt supervised ML algorithm techniques to accurately identify the IoT devices connected to the network. They extracted the network traffic features and fed them into a multi-stage classifier. First, the classifier categorizes whether the traffic belongs to IoT or non-IoT devices. Second, the classifier automatically identifies whether the traffic is generated from an authorized IoT device or not. On the other hand, the goal of authors in [17] was to automatically classify the IoT devices using TCP/IP packets. They proposed a combination of sensor measurement and statistical feature sets in addition to a header feature set using a classification-based device identification framework.

In contrast, in [18], the researchers focus on identifying IoT device types from the whitelist. They collect the traffic of the IoT devices in pcap file format. Then, they extract features of the TCP/IP sessions to train an IoT device-type classifier. In addition, the authors of [19] used header information, sensor measurements, and statistical features to identify the IoT device through profiling each device. Whereas the researchers of [20] used 28 different IoT devices. They collected and synthesized traffic traces; then they identified key statistical attributes (features) to give insights into the underlying network traffic characteristics. They used this dataset and features to develop a multi-stage machine learning-based classification algorithm and demonstrate its ability to identify specific IoT devices.

Based on the previous discussion, it is evident that each literature focuses on particular IoT features to serve specific research objectives. Even though researchers may share a common goal, like identifying abnormal IoT traffic behaviors, they still rely on different IoT network features. This raises a few questions, such as whether these authors have adequately justified their feature selection and what the primary purpose of using specific IoT network features is. Furthermore, it is uncertain whether using the same IoT network features for different purposes will produce comparable outcomes. To address this knowledge gap, our research aims to provide a comprehensive guide or reference for the IoT research community, covering features extracted from any IoT device at both flow and pact levels. Additionally, we aim to develop an automated tool capable of extracting all possible features related to IoT behavior from pre-collected traffic.

**Table 1:** A summary of main IoT studies related to IoT features extraction methods.

Paper Name	Objectives	Method of Implementation	Features
1—Device Identification Based on Communication Analysis for the Internet of Things [15]	Developed a method of device identification that identifies the type and model of devices on the basis of general communication information. It determines the type and model of devices by calculating the similarity of features extracted from their network packets.	(1) Acquisition of communication information: extract header information from the communication packet, which expresses characteristics of devices (2) Extraction of communication features: By adding an element of time, single header information becomes more effective for device identification and intercept of the primary approximate curve within each certain period. The maximum, minimum, and average values express the existence or absence of the burst of the communication packet transmitted and received. (3) calculation of similarity by communication Features: If a device has connected to the network at least once, its communication features are stored in the database. If no type or model has a similarity higher than the certain threshold, the device identifier determines that the target device is a new device in the network.	1—packet length 2—TCP port 3—element of time Within each certain period: 4—the maximum value 5—the minimum value 6—the average value 7—the slope Network layer: 8—packet length 9—time to live (TTL) Transport layer: 10—TCP window size Application layer: 11—HTTP header
2—Behavioral fingerprinting of Internet-of-Things devices [16]	Profiles a device based on information available about the device, generates a robust, verifiable, and unique identity for the device, and establishes some guidelines regarding the device’s operations.	(1) In the first step, the fingerprinting device or tool is strategically placed to capture the relevant information required to fingerprint a device (2) In the second step, the relevant features essential to represent the device fingerprint are either recorded from the information or inferred with the help of transformations like FFT (3) in the fingerprint generation/registration, the fingerprint generation step encodes the features from the previous step and records them against the device’s identifier. Depending on the type of fingerprint recognition algorithm used, this step stores the identified fingerprints differently. (4) Final step, the fingerprint recognition algorithm validates or “reidentifies” a device’s run-time fingerprint against the stored fingerprints with the help of a similarity measurement technique.	1—flow volume, 2—flow duration, 3—average flow rate, 4—device sleep time, 5—number of server ports visited, 6—number of distinct DNS queries, 7—number of NTP queries 8—number of SSL/TLS cipher suites used.
3—ProfilIoT: A Machine Learning Approach for IoT Device Identification Based on Network Traffic Analysis [14]	for accurate identification of IoT devices connected to a network. The goal is to determine whether the traffic belongs to a PC, a smartphone, or a specific (known) IoT device.	(1) The TCP packets were first converted by the feature extractor tool [4] to sessions. (2) Then, each session was represented by a vector of features from the network, transport, and application layers.	1—source and destination IP addresses 2—port numbers from SYN to FIN).
4—Automated IoT Device Identification using Network Traffic [17]	To automatically classify the IoT devices using TCP/IP packets.	the IoT devices using TCP/IP packets ML algorithms (DT, K48, OneR, PART) to classify device type GA to determine the most unique features from the network, transport, and application layer	
5-Detection of Unauthorized IoT Devices Using Machine Learning Techniques [18]	To identify IoT device types from the whitelist.		1—ttl_min: TCP packet 2—time-to-live, minimum 3—ttl_B_min: TCP packet time-to-live sent by server 4—ttl_firstQ: TCP packet time-to-live, quartile 1 5—ttl_avg: TCP packet time-to-live, average 6—ttl_B_thirdQ: TCP packet time-to-live sent by server, quartile 3 7—ttl_B_median: TCP packet time-to-live sent by server, median 8—ttl_B_firstQ: TCP packet time-to-live sent by server, quartile 1 9—ssl_dom_server_name_alexaRank: Alexa Rank of dominated SSL server 10—bytes_A_B_ratio: Ratio between the number of bytes sent and received 11—reset: Total packets with RST flag 12—http_dom_host_alexaRank: Dominated host Alexa rank 13—ttl_thirdQ: TCP packet time-to-live, quartile 3 14—ttl_max: TCP packet time-to-live, maximum 15—ttl_B_var: TCP packet time-to-live sent by server, variance
6—Classifying IoT Devices in Smart Environments Using Network Traffic Characteristics [20]			1—flow volume, 2—flow duration, 3—average flow rate, 4—device sleep time, 5—server port numbers, 6—DNS queries, 7—NTP queries 8—cipher suites
7—Automated IoT Device Identification Based on Full Packet Information Using Real-Time Network Traffic [19]	To identify the device using device profiling	header information, sensor measurements, and statistical features Packet headers were captured using Pyshark, and the nominated header features have been stored in header-DB.	A—Network layer: 1—‘length’ 2—‘time_to_live’ B—Transport layer: 1—‘source_port’ 2—‘stream_index’ 3—‘length’ 4—‘sequence_number’ 5—next_sequence_number’ 6—‘header_length’ 7—‘window_size_value’ 8—‘window_size’ 9—window_size_scalefactor’ 10—‘options’ 11—‘analysis_initial_rtt’ 12—‘analysis_bytes_in_flight’ 13—‘analysis_push_bytes_sent’ 14—‘time_relative’ 15—‘time_delta’ C- Application layer: ‘content-length’ D—Statistics: 1—Flow duration 2—Inter-arrival time

### 2.2. Dataset Collection

This section summarizes the methods used to collect IoT datasets for several objectives.

A recent IoT dataset was released in 2022 by The Canadian Institute for Cybersecurity (CIC). This dataset is used by several sectors, such as universities, private industry, and independent researchers, which can be accessed on the following link: https://www.unb.ca/cic/datasets/iotdataset-2022.html, (accessed on 16 April 2023). The researchers collected network traffic data from IoT devices in six different types of experiments: Power, Idle, Interactions, Scenarios, Active, and Attacks. They used Wireshark and Dumpcap to capture the traffic and conducted experiments to observe device behavior in different scenarios, such as when devices interact with each other or during attacks. The data were collected both manually and semi-automatically [21].

On the one hand, the researchers of [22] used two smart home devices, SKT NUGU, and EZVIZ Wi-Fi Camera, along with other devices connected to the same wireless network. They created various types of network attacks in the IoT environment using tools such as Nmap, except for the Mirai Botnet category, which involved generating attack packets on a laptop. The dataset consisted of 42 raw network packet files captured at different time points using the monitor mode of a wireless network adapter. The files were preprocessed by removing wireless headers using Aircrack-ng. They provide access to the dataset through the following link: https://ocslab.hksecurity.net/Datasets/iot-network-intrusion-dataset, (accessed on 16 April 2023).

Koroniotis et al. [23] created the BoT-IoT dataset in the Cyber Range Lab of UNSW Canberra and includes a combination of normal and botnet traffic on the following link: https://research.unsw.edu.au/projects/bot-iot-dataset, (accessed on 16 April 2023). The dataset’s files are provided in different formats, including pcap, argus, and CSV. The files were separated by attack category and subcategory to aid in labeling. The dataset includes DDoS, DoS, OS, Service Scan, Keylogging, and Data exfiltration attacks. The captured pcap files are 69.3 GB in size with over 72 million records, while the extracted flow traffic in CSV format is 16.7 GB in size. The authors used correlation and entropy to quantify the quality of their dataset. Also, they used several statistical methods along 133 with ML to evaluate the realisticness of the BoT-IoT dataset. Consequently, they provide a baseline for 134 botnet identification across IoT-specific networks.

Research conducted by Stratosphere Laboratory, as mentioned in [24], has resulted in the creation of an IoT-23 dataset that focuses on network traffic from IoT devices. This dataset has been specifically designed to aid in the detection of IoT-based botnets. The objective of this dataset is to provide researchers with a sizable collection of real and labeled IoT malware infections as well as IoT benign traffic. The dataset was published in January 2020 and included a total of 20 malware capture traffic and three captures of benign IoT traffic; the dataset can be accessed on the following link: https://www.stratosphereips.org/datasets-iot23, (accessed on 16 April 2023).

A new automated toolchain, called CREAM, has been created by the developers of [25]. This toolchain combines various tools to automate the whole process of configuration, attack and benign behavior reproduction, data collection, feature extraction, data labeling, and evaluation. The toolchain has made it possible to create a reliable dataset, which has been evaluated for its quality and efficiency. Furthermore, it has the ability to collect and produce data from various sources, such as accounting, network traffic, and system logs. On the other hand, [26] authors have provided a framework to develop a dataset for intrusion detection system evaluation and testing. This dataset is useful for IoT researchers as it provides a relevant DDoS dataset to test their models developed to counter DDoS attacks. In addition, users can regenerate the dataset as and when required, as the developed framework can be used to collect data at any time. As a result, this dataset is self-sustainable.

This research [27] offers a valuable contribution—a labeled behavioral IoT data set. This data set comprises normal traffic and malicious network traffic generated by botnets in a medium-sized IoT network infrastructure. The data was collected from three prominent botnet malware—Mirai, BashLite, and Torii, including information on botnet infection, propagation, and communication with C&C stages. The binary and multi-class machine learning classification models run on the acquired data demonstrate that the generated data set is suitable and reliable for machine learning-based botnet detection IDS testing, design, and deployment. The IoT behavioral data set is now publicly available as a MedBIoT data set. In addition, the contribution of this.

As a result, we noticed that the first step in any IoT research is analyzing its network traffic. However, there is no standard method of doing such a process, which motivated us to create a public and holistic IoT traffic analyzer tool that serves any IoT research.

## 3. Methodology

This section describes the methods used to build the innovative public IoT analyzer tool. Section 3.1 explains the framework environment used in this research, the network configuration as well as the setup of the smart home IoT devices. While Section 3.2 discusses the methods used to collect the IoT traffic from 4 IoT devices, as well as details the process of analyzing and extracting any IoT device type, and finally explains the proposed tool’s creation and implementation.

### 3.1. Framework Environment

Extracting network features and creating a dataset in any context requires careful collection framework design to guarantee accurate and unbiased results, which in turn requires pre-planning and thinking out how every aspect of the network should be designed. According to [27], there are several methods to set up a smart home environment to collect and monitor traffic. The first method is by configuring a switch for “Port Mirroring/Port Spanning”, while the second method is by using OpenWRT on the wireless router, then using “iptables” and “ebtables” for Layer 3 and Layer 2. The third method of collecting and monitoring the traffic is by implementing ARP spoofing; in this case, the whole traffic goes over a sniffing machine. Finally, the last method is configuring a device to work as a gateway or a hot spot, then connecting other devices to this gateway.

In this research, we select the last method because the home gateway provides a central vantage point to measure the characteristics of all devices in a smart home. As a result, we can passively monitor network traffic generated by smart home IoT devices in smart homes as they connect to their cloud services and other third-party services on the Internet. Therefore, our method is low-cost and can commonly be used for various devices.

#### 3.1.1. Network Configuration

**Figure 1:**
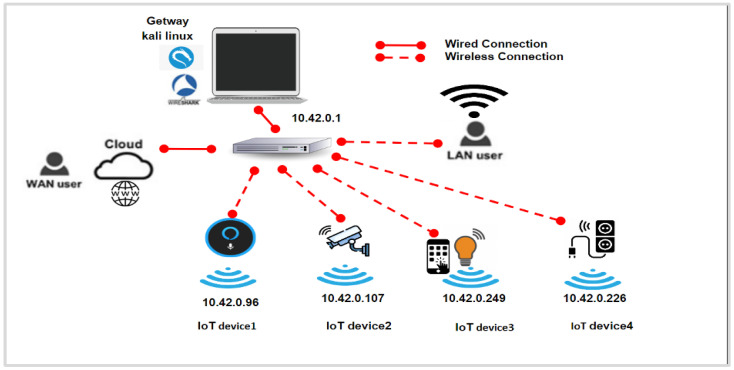
IoT framework environment.

In the framework overview depicted in Figure 1, we first set up the Kali Linux laptop as a gateway to connect the IoT devices to the Internet. This involves connecting the laptop to the router via an Ethernet cable to access the Internet and then activating its Wi-Fi hotspot [28], as outlined in step 2. We proceed to install the recommended app for each IoT device on the Android smartphone and connect the smartphone to the Internet using the Kali hotspot. Finally, we configure the IoT devices to connect to the Internet through the Kali hotspot using their respective IoT apps. With this configuration in place, we can collect and monitor the network traffic between the IoT devices and the Android application to the IoT cloud and vice versa.

#### 3.1.2. Smart home IoT devices

Table 2 presents the IoT devices used to collect real-world smart-home traffic in order to analyze their traffic and extract their features. We captured the communication between the IoT device and its cloud server and between the IoT device and other IoT devices within the network.

#### 3.1.3. Installation Description

**Figure 2:**
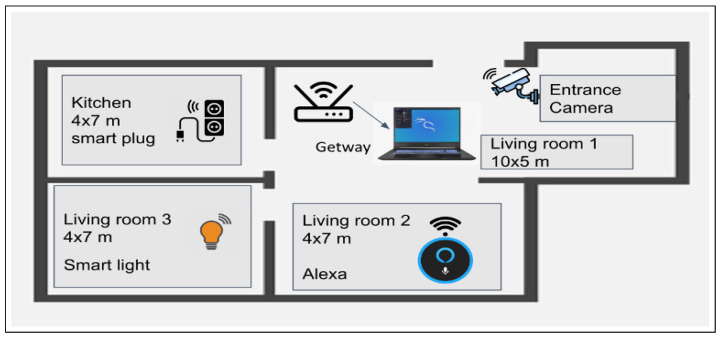
Smart home devices’ installation.

Figure 2 illustrates the plane of the smart home installation by depicting the layout of installed multiple IoT devices in five main sections. All IoT devices are connected to the network via the gateway and communicate differently depending on the type and usage of the device. The distribution of the IoT devices is as follows:

- The home entrance contains a smart camera with a motion sensor to detect movement.
- The living room1 (10 × 5) contains the gateway, providing the Internet to all IoT devices.
- The living room2 (4 × 7) contains Amazon Alexa Eco, which controls the other IoT devices.
- The living room3 (4 × 7) contains the smart light.
- The kitchen (4 × 7) contains a smart plug connected to a boiler.

### 3.2. Smart-Home Traffic Data Collection

This section covers the experimentations used to capture and collect the raw traffic from the IoT devices in several scenarios. We started logging the network traffic in our smart home environment from 10 September 2022 until 30 November 2022. According to the proposed framework, all the traffic of the IoT devices coming through the gateway is automatically collected using the Wireshark tool running on the Kali Linux laptop. Each raw trace data contains packet headers and payload information. In this research, we aim to collect and monitor the benign traffic of IoT devices in several scenarios. Hence, extract the feature and generate a dataset

#### 3.2.1. Scenarios for Collecting Benign Traffic

For collecting and generating real-world IoT datasets, we must understand the normal behavior of the IoT device (i.e., not under attack) in all its situations. To do this, we performed different scenarios for each IoT device individually. All the devices in our environment were powered on, i.e., connected to the socket, and the network traffic was captured in isolation for a duration of 30 min. In addition, we install the recommended apps that control the IoT device in a smartphone. However, it is important to highlight that Alexa has different scenarios than the other three IoT devices due to its functionality, as explained earlier in Table 2.

All the experiments can be organized as follows:

1. Power is on (true/false): In this experiment, we capture the network traffic of the IoT device in two states, i.e., when the device is powered on and when the device is powered off. In addition, we capture the traffic while the user alters between power on and off the device simultaneously. The whole network communications were captured throughout the day.
2. Application is on (true/false): In this experiment, we capture the network traffic of the app that controls the IoT device in two states, i.e., when the app is active (i.e., open) and when the app is inactive (i.e., closed).
3. Device/Application Idle (true/false): In this experiment, we captured the network traffic from the IoT device or the IoT application when it’s either on for a long period as well as no human interactions are involved, which we call idle time. Otherwise, the device or the IoT application is not idle, i.e., human interactions are involved. However, if the IoT device or application was switched off, we call it not applicable (NA).
4. User interaction with the IoT app (true/false): In this experiment, we perform all possible interactions with the IoT device functionality through its app (the app is open and not in an idle state) to generate network traffic. The network activity for each functionality was captured either passively or actively.
5. Play music (true/false): In this experiment, we command Alexa to search for particular music and then play it.
6. Connect to a website (true/false): This experiment is an audio search command. We asked Alexa to answer particular questions like what the weather is, what the news is, what is the capital of a country, etc.
7. Connect to other IoT devices (true/false): a smart plug, smart camera, and smart lamp) to control them. For example, we command Alexa to switch on/off the smart plug.

We conducted different scenarios for each IoT device using a combination of the previous experiments inside a smart home. Note that each device has different scenarios based on its functions. These experiments were done to monitor how devices behave in the network from two perspectives: (1) while communicating with their cloud server/s and (2) while interacting with other IoT devices simultaneously. For example, in Scenario 1, we collected the traffic of the smart plug, which includes opening the app, then powering on the plug through its app. After that, we kept the app open and the plug on for a while without interaction. In total, we implemented seventeen (17) different scenarios collected from four IoT devices; The rest of the scenarios for each IoT device are explained in Table 3 and Table 4. The resulting traces were stored as pcap files for analysis and feature extraction in the storage device.

#### 3.2.2. The Proposed Tool (IoT TAHFE)

**Figure 3:**
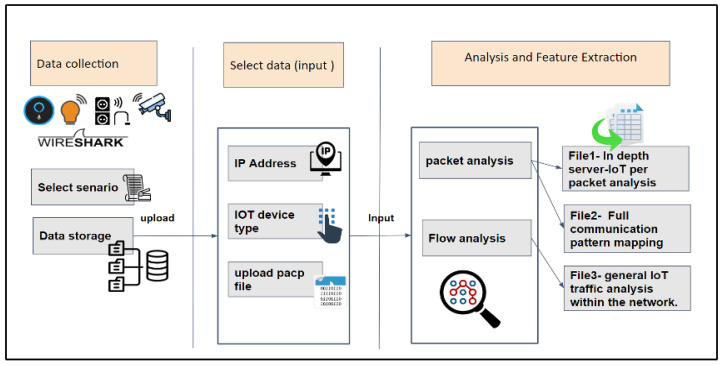
Overview of the proposed IoT TAHFE tool that holistically analyzes and extracts the features at flow and packet level.

Figure 3 presents a high-level overview of the IoT Traffic Analyzer Tool with Automated and Holistic Feature Extraction Capability (IoT TAHFE). As mentioned earlier, the objective of the IoT TAHFE tool is to automatically analyze the pcap file and profile of the IoT device’s normal behavior by extracting all the network features (i.e., network packets and network flow). Thus, this research considers the first one that aims to support IoT researchers and speed up their research. Our tool is now available to all IoT researchers through the following link: http://iottrafficanalyzer.com/, (accessed on 15 May 2023). Also, we upload the source code of the tool on our GitHub page on the following link: https://github.com/Malmasre/TAHFE_IOTtrafficAnalyzer, (accessed on 15 May 2023).

**Figure 4:**
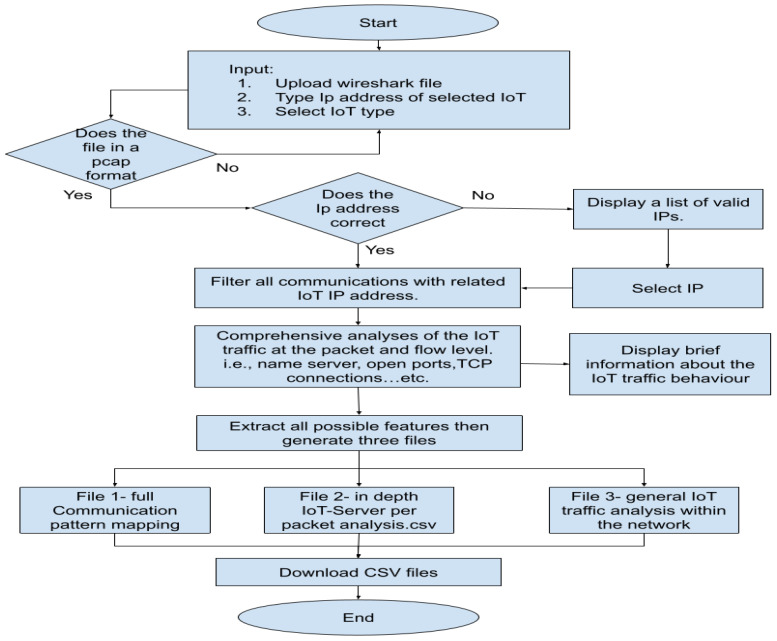
Overview of the algorithm used to analyze and extract the IoT features from pre-collected traffic in a pcap format.

To test the tool, we utilized pre-collected IoT traffic from one of the devices we used, i.e., the smart plug, using one of the scenarios outlined in Section 3.2.1. First of all, we navigate to the IoT TAHFE tool on the webpage mentioned above. An overview of the steps our algorithm takes to analyze and extract the potential features of an IoT device can be seen in Figure 4. The tool will ask the user to enter three required parameters as input:

1. Provide the IP address of the IoT device for deep analysis.
2. Select the IoT device type from a drop-down list (i.e., smart camera, smart plug, etc.).
3. Upload the pcap file of the IoT device.

After that, the tool will automatically analyze the pcap file and profile of the IoT device’s normal behavior by extracting all the network features (i.e., network packets and network flow). Finally, it will generate three different CSV files, each of which has different features. See the detailed steps of implementing the IoT TAHFE tool in Appendix A.

**Figure 5:**
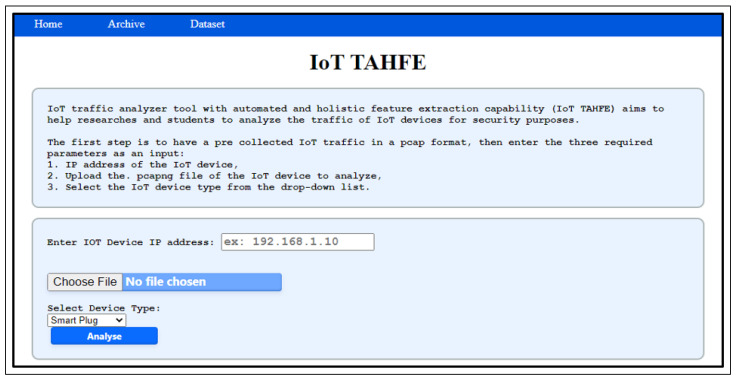
The IoT TAHFF homepage, which represents the first Stage that requires three inputs.

For example, The first stage is to provide the tool with the required inputs. In our case, we provide the Ip address of the smart plug. Figure 5 illustrates the initial screen where the user can input the three main values mentioned above.

Notice that if the device type is not in the list, the user can choose another one and write the device type. Also, the user can analyze one pcap file at a time.

The second stage is to check the validity of the inputs. In this stage, the tool will run several checkups:

1. check whether the uploaded file is in pcap file format or not. Otherwise, it will give an error message to upload the correct file format.
2. check whether the IP address is typed correctly; otherwise, an error message appears to inform the user to enter the correct IP format.

In the third stage, the user is ready to analyze the pcap file by clicking on the “Analyze” button. However, during the analysis time, another essential checkup happens. The tool ensures that the IoT IP address entered by the user is included in the pcap file. Otherwise, the tool returns a clickable list of valid IPs which are not server IP addresses. Then, the tool will select the last valid IP from the IP list by default; however, the user can edit the IP address by selecting the required one from the list or typing it manually. Once the user types the correct IP address, the file needs to be uploaded again, then click on the “Analyze” button to start the analysis process.

**Figure 6:**
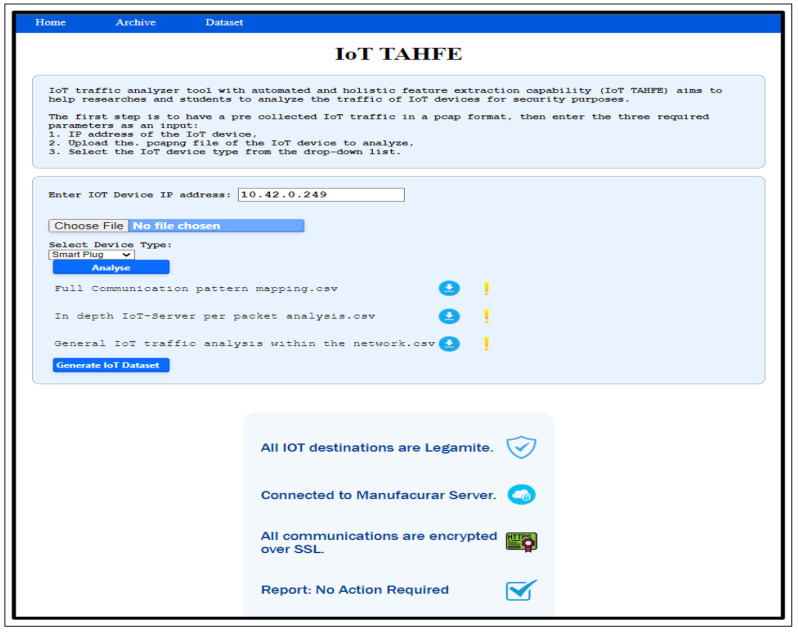
The last stage of the tool where the analysis of the pcap file finished and the generated files can be downloaded by the user.

At the last stage, As shown in Figure 6, the tool analyzes the pcap file in-depth according to the selected IoT IP address. As a result, three different CSV files are generated, each of which has different features as well as different network properties. The user can download the files by clicking on the download icon in front of each file. The description of each file will be explained separately in the next Section 4. Finally, the entire analysis history can be viewed, downloaded, and deleted from the archive tap.

## 4. Result and Discussion

This section discusses the results of implementing the tool on a selected pcap file to analyze the traffic and extract the feature for the row data collected from the previous scenarios. Also, the evaluation of implementing the tool is discussed in Section 4.3.

To understand the communication pattern of a specific IoT device, between two or more IoT devices, or between a particular IoT device and its cloud server, we need to implement a network traffic analysis technique. According to [29], “Traffic analysis is the process of capturing and examining network data in order to deduce information from patterns in communication”. In general, the more data that you capture, the more you can infer from the traffic. In this research, we mainly focus on extracting features that help IoT researchers to identify the following:

1. The IoT device type, i.e., smart light, smart camera, etc.
2. The IoT device behavior, i.e., whether the device is on, of, or idle.
3. Human interaction type, i.e., determine the type of interaction between the user and the IoT device or the IoT app. For example, if the user is using the IoT app to switch on or off the smart plug.
4. IoT behavior within the network, i.e., if the IoT interacts with other IoT or non-IoT devices that are connected to the same network.
5. Abnormal behavior, i.e., attacks such as MITM detection or IP address spoofing detection.

The tool reads each packet from the pcap file individually and then extracts its contents. The extracted information includes important fields from the packet header along with metrics, such as the packet’s size. To perform traffic analysis on the network, two main technologies can be chosen; flow analysis and packet analysis. In contrast to previous research, which focused solely on analyzing either IoT packets or IoT flow. This research aims to analyze IoT traffic more broadly and comprehensively by analyzing IoT packets and traffic flow as follows. Therefore, we analyze the IoT traffic deeply by implementing two phases; Phase one is Packet analysis, and Phase two is Flow analysis.

### 4.1. Phase One: Packet Analysis Level

The packet analysis focus aims to uncover what is contained within packet payloads. Hence the IoT TAHFE tool aims to apply deep packet inspection (DPI) technologies that use packets as a data source and then extract all metadata. As a result, two CSV files were generated, In-depth IoT-Server per packet analysis.csv and Full Communication pattern mapping.csv.

The first generated CSV file contains all the features extracted from analyzing every packet individually between the IoT device and the server/s or between the IoT device and other IoT devices; in total, we have 24 features. Table 5 demonstrates each extracted feature in detail as follows:

- First column “Feature”: list all the feature’s name.
- Second column “Description”: describe the purpose of the feature and its role in the packet network.
- Third column “Usage”: explain the objectives behind using such a feature.
- Fourth column “Importance”: explain the importance of using this feature or, in other words, why it is important in terms of the research.
- Fifth column “Network layer”: specify which OSI (Open Systems Interconnection) layer belongs to such a feature.

**Table 5:** Features Extracted from in depth IoT-Server per packet analysis.

No.	Feature	Description	Usage	Importance	OSI Layer
1	Dest_IP	destination IP addresses that communicate with the IoT device.	Each IoT connects with a fixed set of IPs that rarely change except for streaming devices.	Identify the IoT device type.	Transport layer and IP Layer.
2	Dest_port_ no	determines the port number of the destination IP address communication.	There is a usual range of communications ports for each device.	Identify the IoT device type	Transport layer. TCP/UDP Layer.
3	IoT_port_no	Determine the port number on the IoT device that communicates with the destination IP address.e.g., port 9999	There is a usual range of communications ports for each device.	1. Identify the IoT device type 2. Identify the IoT device behavior	Transport layer. TCP/UDP Layer.
4	Protocol	The application protocol of the communication. For example DNS & FTP & HTTP & IMAP & etc.	For each destination IP address & determine the protocol of the communication with the IoT device.	1. Identify the IoT device type 2. Identify the IoT device behavior	Transport layer. IP Layer.
5	Send_ receive_ ratio	Calculating the ratio of the number of packets sent from the IoT device divided by the number of packets received from the server per minute in bytes	represents a pattern of sending to receiving	1. Identify the IoT device type 2. Identify the IoT device behavior 3. Identify human interaction type 4. Identify abnormal behavior	Transport layer. TCP/UDP Layer.
6	No_of_ received_ packets_per_ minutes	Calculating the number of packets received by the IoT device per minute.	In normal usage & the number of received packets is in a specific range.	1. Identify the IoT device type 2. Identify the IoT device behavior 3. Identify human interaction type 4. Identify abnormal behavior	Transport layer. TCP/UDP Layer.
7	No_of_sent_ packets_per_ minutes	Number of packets sent from the IoT device per minute	In normal usage & the number of sent packets is in a specific range.	1. Identify the IoT device type 2. Identify the IoT device behavior 3. Identify human interaction type 4. Identify abnormal behavior	Transport layer. TCP/UDP Layer.
8	Avg TTL	Calculating the average of the maximum hops needed by a message to reach the server from the IoT device.	Each server usually has a const number of TTL.	Identify abnormal behavior	Data Link layer. IP Layer.
9	Flow volume	Calculating the total of download and upload bytes	Used to calculate the flow rate.	1. Identify the IoT device behavior 2. Identify human interaction type	Transport layer. TCP/UDP Layer.
10	Flow duration	Calculating the time (in minutes) between the first packet and the last packet in a flow.	Used to calculate the flow duration.	1. Identify the IoT device behavior 2. Identify human interaction type	Data Link layer. IP Layer.
11	Dest_ip_avg _packet _length	The average of the packet’s length, including the headers received by the destination IP from the IoT device.	IoT—API communications usually have a set of fixed packet length.	1. Identify the IoT device type 2. Identify the IoT device behavior 3. Identify human interaction type	Network layer. IP layer.
12	Src_ip_avg _packet _length	The average of the packet’s length, including the headers sent by the destination IP to the IoT device.	IoT—API communications usually have a set of fixed packet length.	1. Identify the IoT device type 2. Identify the IoT device behavior 3. Identify human interaction type	Network layer. IP layer.
13	Flow_rate	Calculating flow volume divided by the flow duration (in seconds)	IoT devices have a set of bandwidth values for each state.	1. Identify the IoT device behavior	Transport layer. TCP/UDP Layer.
14	IsServer	Determine whether the destination IP address is a server or not	This feature is important to identify if the IoT device communicates with other IoT devices or with the server	1. Identify the IoT device type	Network layer. IP layer.
15	Max_dest_ SSL_ payload	The maximum payload size of the SSL sent from the destination IP address to the IoT device.	Communication pattern recognition	1. Identify the IoT device behavior 2. Identify the IoT device behavior 3. Identify human interaction type	Network layer. SSL - UDP - HTTP layer.
16	Min_dest_ SSL_ payload	The minimum payload size of the SSL sent from the destination IP address to the IoT device.	Communication pattern recognition	1. Identify the IoT device behavior 2. Identify the IoT device behavior 3. Identify human interaction type	Network layer. SSL - UDP - HTTP layer.
17	Avg_dest_ SSL_ payload	Calculate the average size of the SSL payload sent from the destination IP address to the IoT device.	Communication pattern recognition	1. Identify the IoT device behavior 2. Identify the IoT device behavior 3. Identify human interaction type	Network layer. SSL - UDP - HTTP layer.
18	Std_dest_ SSL_ payload	Calculate the standard deviation size of the SSL payload sent from the destination IP address to the IoT device.	Communication pattern recognition	1. Identify the IoT device behavior 2. Identify the IoT device behavior 3. Identify human interaction type	Network layer. SSL - UDP - HTTP layer.
19	Max_IoT_ SSL_ payload	The maximum size of the SSL payload sent from the IoT device to the destination IP address IoT device.	Communication pattern recognition	1. Identify the IoT device behavior 2. Identify the IoT device behavior 3. Identify human interaction type	Network layer. SSL - UDP - HTTP layer.
20	Min_IoT_SSL_ payload	The minimum size of the SSL payload sent from the IoT device to the destination IP address IoT device.	Communication pattern recognition	1. Identify the IoT device behavior 2. Identify the IoT device behavior 3. Identify human interaction type	Network layer. SSL - UDP - HTTP layer.
21	Avg_IoT_ SSL_ payload	Calculate the average size of the SSL payload sent from the IoT device to the destination IP address.	Communication pattern recognition	1. Identify the IoT device behavior 2. Identify the IoT device behavior 3. Identify human interaction type	Network layer. SSL - UDP - HTTP layer.
22	Std_IoT_ SSL_ payload	Calculate the standard deviation size of the SSL payload sent from the IoT device to the destination IP address.	Communication pattern recognition	1. Identify the IoT device behavior 2. Identify the IoT device behavior 3. Identify human interaction type	Network layer. SSL - UDP - HTTP layer.
23	Dest_TCP_ Flags	The flag type of the packet in the TCP layer send from the IP destination to the IoT device	determines the flag type from SYN to ACK	1. Identify the IoT device type 2. Identify abnormal behavior	transport layer. Layer TCP
24	IoT_TCP_ Flags	The flag type of the packet in the TCP layer sends from the IoT device to the IP destination.	determines the flag type from SYN to ACK	1. Identify the IoT device type 2. Identify abnormal behavior	transport layer. Layer TCP

The second extracted CSV file (Full Communication pattern mapping.csv) is different than the first one in terms that it focuses on analyzing the communication pattern of a particular IoT device. Each IoT device has a different communication method; hence, the researcher will be able to identify the device type as well as identify a particular activity of such a device. Consequently, each row in the CSV file represents the extracted features of the packets that form one complete communication between the IoT device and its cloud server or between the IoT device and other IoT devices. Such communication is determined based on the acknowledgment number and the next sequence number (from syn to fin). Noting that this analysis only works with TCP communications, not UDP. Table 6 demonstrates the extracted feature in detail as follows:

- First column “Feature”: list all the features name of that represent one complete communication.
- Second column “Description”: describe the purpose of the feature and its role in the packet.
- Third column “Usage”: explain the objectives behind using such a feature.
- Fourth column “Importance”: explain the importance of using this feature or, in other words, why it is important in terms of the research.

**Table 6:** The extracted features from analyzing the Full Communication pattern mapping.

No.	Feature	Description	Usage	Importance
1	src_IP	IP of the device that initialized the communication (either the IoT or the server)	Communication Analysis. Identifies the type and model of devices. Profile the device.	Identify the IoT device type
2	src_Port	Port number of the device that initialized the communication either from the IoT device or the IoT server	Communication Analysis. Identifies the type and model of devices. Profile the device.	Identify the IoT device type
3	dst_IP	IP address of the device that received the communication either from the IoT device or the IoT server	Communication Analysis. Identifies the type and model of devices. Profile the device.	Identify the IoT device type
4	dst-port	Communication port of the device that received the communication either from the IoT device or the IoT server	Communication Analysis. Identifies the type and model of devices. Profile the device.	Identify the IoT device type
5	Protocol	communication protocol (only TCP) either from the IoT device or the IoT server	Communication Analysis. Identifes the type and model of devices. Profile the device.	Identify the IoT device type
6	req_packet_ Length	total size of the packet (including headers) of the request packet either from IoT device or IoT server	Communication Analysis. Identifies the type and model of devices. Profile the device.	1. Identify the IoT device type 2. Identify the IoT device behavior 3. Identify human interaction type
7	res_packet_ Length	total size of the packet (including headers) of the request packet either from the IoT device or the IoT server	Communication Analysis. Identifes the type and model of devices. Profile the device.	1. Identify the IoT device type 2. Identify the IoT device behavior 3. Identify human interaction type
8	req_Payload	The size of the actual data (excluding headers) Of the request packet either from the IoT device or the IoT server	Communication Analysis. Identifes the type and model of devices. Profile the device.	1. Identify the IoT device type 2. Identify the IoT device behavior 3. Identify human interaction type
9	resp_packet_ Payload	The size of the actual data (excluding headers) Of the response packet either from the IoT device or the IoT server	Communication Analysis. Identifes the type and model of devices. Profile the device.	1. Identify the IoT device type 2. Identify the IoT device behavior 3. Identify human interaction type
10	req_TTL	request Time-to-live (TTL) is a value for limiting the maximum period of time that a packet should exist on the network before being discarded.	communication pattern recognition. Each server communication usually has a single value for all communication.	Identify abnormal behavior i.e., attacks such as MITM detection or IP address spoofing detection
11	resp_time_min	minimum response time (difference between request and response time as recorded by the capturing device) for that communication pattern.	communication pattern recognition. If time is below minimum time, it is likely that the server is closer now.	Identify abnormal behavior i.e., attacks such as MITM detection or IP address spoofing detection
12	resp_time_avg	average means summing all communication responses times as defined above and dividing the sum by their count.	communication pattern recognition. Communication Analysis.	Identify abnormal behavior i.e., attacks such as MITM detection or IP address spoofing detection
13	resp_time_max	Maximum response time (difference between request and response time as recorded by the capturing device) for that communication pattern.	communication pattern recognition. If time is above maximum time, it is likely that there is slow internet detection.	Identify abnormal behavior i.e., attacks such as MITM attack have been established and data is delayed to being proceeded by a new node and passing through a longer route including the MITM device.
14	repentance	The number of times that this communication pattern repeated in the pcap file.	communication pattern recognition. The more this communication has repeated the more it is likely it represents a normal usage or an API communication.	1. Identify the IoT device type 2. Identify the IoT device behavior 3. Identify human interaction type
15	Repentance_ per_minute	The number of times that this communication pattern repeated per minute.	communication pattern recognition. The more this communication has repeated the more it is likely it represents a normal usage or an API communication.	1. Identify the IoT device type Identify current management status (if the device is being controlled by app).
16	IsServer	does one of the communication sides have a valid server name.	Communication Analysis. Devices from which hackers launch their attacks are very unlikely to have a valid server name as it requires payment which can be linked to the user. (some hosting sites allow untraceable payments by Bitcoin and Ethereum)	1. Identify the IoT device type Identify current management status (if the device is being controlled by the app).

### 4.2. Phase Two: Flow Analysis Level

Unlike packet analysis, flow analysis gives a statistical summary or high-level look at network statistics regarding the device behavior within the network. Flow analysis helps detect anomalies in traffic behavior that could indicate security breaches by identifying the infected patterns within the network [30]. Each flow represents a session between the two hosts. Table 7 demonstrates 15 statistical features calculated from the fields and payload of the packets contained in the flow as follows:

- First column “Feature”: list all the features names that represent one complete communication.
- Second column “Description”: describe the purpose of the feature and its role in the packet.
- Third column “Usage”: explain the objectives behind using such a feature.
- Fourth column “Importance”: explain the importance of using this feature or, in other words, why it is important in terms of the research.

**Table 7:** Features extraction from general IoT traffic analysis within the network.

No.	Feature	Description	Usage	Importance
1	Sum_of_all_ packets	calculates the total number of all sent and received packets per minute that targeted the IoT device.	to determine the total number of packets in normal communication. However, a change in number of packets may requires further inspection.	Identify abnormal behavior
2	No_TCP_ handshake	calculates the total number of TCP handshakes between the IoT device and the destination IP address in a traffic flow	to determine the total number of handshakes in normal communication. However, a change in the number of handshakes may require further inspection.	Identify abnormal behavior
3	TCP_reset	calculates the total number of rejected connections by the IoT device.	to determine the total number of packets sent by the IoT that contain a set-reset flag in normal communication.	Identify abnormal behavior, i.e., if someone is trying to implement reconnaissance against the IoT device.
4	No_of_IP_ servers	Total number of IP servers that communicate with the IoT device	to determine the total number of independent servers that communicate with the IoT; either the IoT servers or any third-party servers	Identify the IoT behavior within the network, i.e., if the IoT device communicates with other IoT devices or with the server.
5	No_of_TCP_ connection	Number of TCP connection in normal network flow	to determine the total number of TCP connections that was established with the IoT. Each IoT has max of 1 or 2 TCP connections. extra connections indicate that the device is being accessed by multiple users.	1. Identify the IoT behavior within the network 2. Identify abnormal behavior
6	No_UDP_ connection	Number of UDP connections in normal network flow	to determine the total number of UDP connections that was established with the IoT. Each IoT has a max of 2 UDP connections except streaming connections. extra connections indicate that the device is being accessed by multiple users.	1. Identify the IoT behavior within the network 2. Identify abnormal behavior
7	No_of_Dest_ TCP_ports	Total number of destinations TCP ports in a normal network flow	to determine the total number TCP ports, open on the remote server. Usually IoT connect to APIs on 80 and 443, new ports would require inspection.	1. Identify the IoT behavior within the network 2. Identify abnormal behavior
8	No_IoT_TCP_ ports	Total number of open IoT TCP ports in a normal network flow	to determine the total number of open TCP ports on the IoT device. IoT has a range of ports to start TCP connections.	1. Identify the IoT behavior within the network 2. Identify abnormal behavior 3. Identify the IoT device type
9	No_of_Dest_ UDP_ports	Total number of destinations UDP ports in a normal network flow	to determine the total number of UDP ports open on remote servers. Usually IoTs connect to time and DNS servers on 123 and 53	1. Identify the IoT behavior within the network 2. Identify abnormal behavior 3.Identify the IoT device type
10	No_of_IoT_ UDP_ports	Total number of open IoT UDP ports in a normal network flow	to determine the total number UDP ports open on the IoT. IoT has a range of ports to start UDP connections.	1. Identify the IoT behavior within the network 2. Identify abnormal behavior 3. Identify the IoT device type
11	No_of_ irresponsive_ ports	Number of ports that do not respond to a connection	to determine the total number of ports that didn’t respond back to the communication attempt. Irresponsive ports are a definite indication of exploitation.	Identify abnormal behavior
12	No_of_DNS_ request	Number of DNS queries in a normal network flow	to determine the total number of DNS requests initialized by the IoT. Connections reset are usually followed by DNS requests and NTP requests which usually occur before attacks.	Identify abnormal behavior
13	No_of_DNS_ response	Number of DNS responses in a normal network flow	to determine the total number of DNS responses received by the IoT.	Identify abnormal behavior
14	No_of_NTP_ request	Number of time requests in a normal network flow	to determine the total number of NTP requests initialized by the IoT.	Identify abnormal behavior
15	No_of_NTP_ response	Number of time requests in a normal network flow	to determine the total number of NTP responses received by the IoT.	Identify abnormal behavior

### 4.3. Tool Evaluation

In this research, we have developed a comprehensive framework for collecting traffic from IoT devices in various possible scenarios. However, researchers have the freedom to collect IoT network traffic in their preferred way that suits their research. The tool’s functionality is not meant to cover “all scenarios”; instead, its main objective is to assist researchers by saving their time and effort. To achieve this, the proposed tool takes previously collected traffic, whether using one of the mentioned Section 3.2.1 or any method preferred by the researcher, and analyzes it in-depth. As a result, it extracts all possible features at the flow and packet level and creates three CSV files. It is worth noting that the tool analyzes and extracts only the features related to such IoT device behavior, i.e., it is not necessary to extract all the features mentioned in Table 5, Table 6 and Table 7. Yet, IoT researchers can benefit from these tables to explore more information about the features extracted after analyzing their IoT devices. Such tables offer a comprehensive guide and reference that describe each feature and how to use it in research as well as rank the importance of the extracted features from the IoT device based on five different aspects.

**Figure 7:**
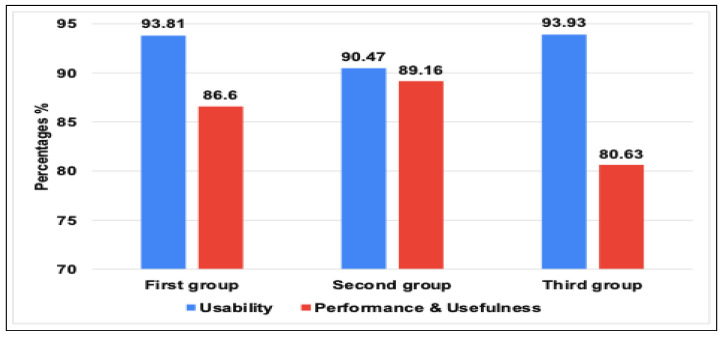
The evaluation results in a percentage of the tool’s usability and performance among the three IoT researchers groups.

**Figure 8:**
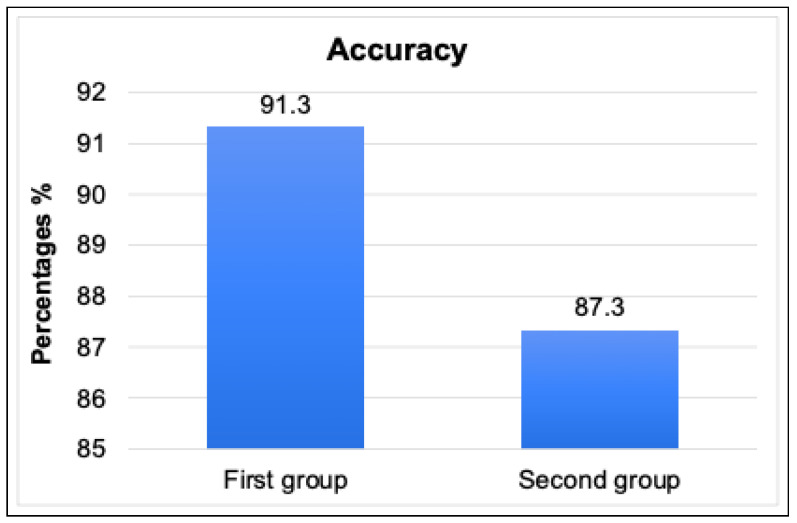
The evaluation result in a percentage of the tool’s accuracy among two IoT researchers groups (one and two).

In order to ensure the usefulness, accuracy, performance, and usability of our IoT TAHFE tool, we conducted a series of various evaluations. Thus, we enlisted the help of 20 participants, including five IoT researchers, four Ph.D. students, six master students, and five bachelor students. Then, we divided the participants into three groups based on their level of IoT research. The first group included the IoT researchers who had already completed their research and published their findings. The second group included the IoT researchers who were still in the process of collecting and analyzing IoT traffic, while the third group included the IoT researchers who had yet to begin their research on IoT traffic analysis. Noting that each group had different research contributions, such as identifying security vulnerabilities, classifying IoT devices, and determining the behavior of IoT devices. After that, we asked each group to apply our tool in their research and compare its output to the results they had previously obtained. In addition, we asked all participants to provide feedback on the tool’s usefulness, accuracy, performance, and usability. A list of questions can be found in Appendix B. We summarized the collected results from the feedback we received and divided them according to three metrics, i.e., percentage, mean, and standard deviation. On the one hand, the results in a percentage of the proposed tool among the three IoT researcher groups can be concluded as follows:

1. As seen in Figure 7, all three groups of IoT researchers expressed high satisfaction levels with the tool’s interface and ease of use (usability of the tool). The first and second groups reported scores of 93.8%, while the last group had a score of 90.5%. These results indicate that users found the tool easy to use and had a positive overall experience while interacting with it.
2. We assessed the tool’s performance and usefulness based on the perceptions of the three IoT researcher groups. We tested the tool’s speed of extracting all possible features from network traffic data and its usefulness in providing meaningful information that researchers can use to improve IoT systems’ security and efficiency. All three groups expressed their satisfaction with the performance and usability of the tool, with scores of 86.6%, 89.16%, and 80.36%, respectively.
3. We evaluated the accuracy and relevance of the information provided by the tool. As you can see in Figure 8, we relied on the perceptions of the first and second groups of researchers, who already had results to compare with. This involved assessing how well the tool can extract relevant information from network traffic, such as identifying IoT devices, behavior patterns, human interactions, and abnormal behavior data, and how reliable the tool is. The first group found the extracted features to be very similar to their published results, with a rate of 91.3%, while the second group rated the accuracy moderately similar with 87.3%.

**Figure 9:**
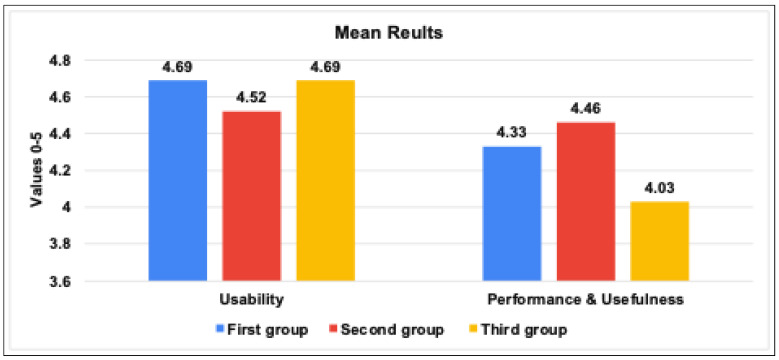
The evaluation of the mean results of the tool’s usability and performance among the three IoT researchers groups.

**Figure 10:**
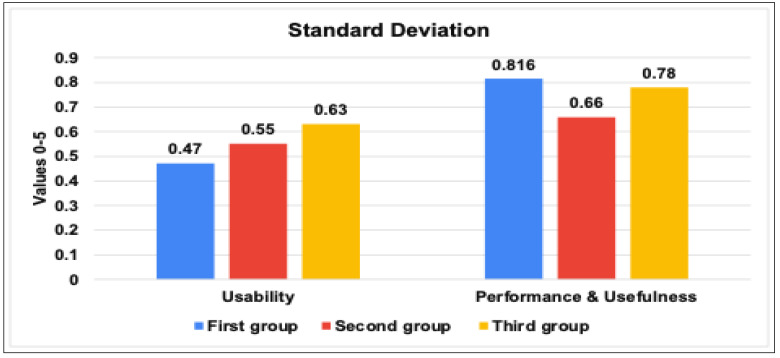
The evaluation of the standard deviation results of the tool’s usability and performance among the three IoT researchers groups.

**Figure 11:**
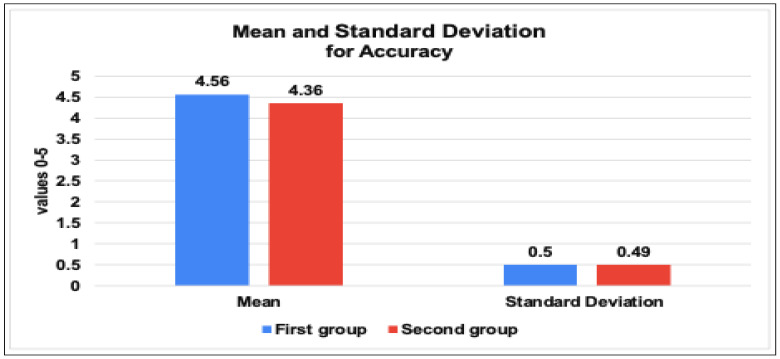
The evaluation of the mean and standard deviation results of the tool’s accuracy among two IoT researchers groups (one and two).

On the other hand, the results in mean and standard deviation of the proposed tool among the three IoT researcher groups can be concluded as follows:

1. Figure 9 demonstrates the results of the mean values for the usability, usefulness & performance of the tool among the three IoT research groups. The study found that users generally view the IoT traffic analyzer tool as highly usable, with an average score ranging from 4.52 to 4.69 across three groups of users. The first and third groups rated the tool consistently high in both usability and performance, while the second group gave slightly lower scores but still found the tool useful. Overall, the tool and benchmark framework developed are seen as valuable for measuring IoT network behavior in certain industries.
2. Figure 10 demonstrates the results of the standard deviation values for the usability and performance of the tool, which provides insights into the variability of scores obtained by three groups of users. Usability has a low standard deviation range, indicating consistency across all three groups of users. Performance shows slightly higher variability, with the second group having the highest standard deviation. However, the standard deviation values suggest that the tool is consistent and provides stable results, particularly in its usability. These results suggest that the IoT traffic analyzer tool and benchmark framework can be useful for IoT researchers in various industries.
3. Figure 11 found that the user’s perception of the accuracy values for the tool is positive across the two groups. The first group gave a higher average score of 4.56 with a standard deviation of 0.5, and the second group gave a slightly lower average score of 4.36 with a slightly lower standard deviation of 0.49.

Overall, the results suggest that users find the tool to provide reliable and accurate analysis of IoT network traffic. The consistency of the scores, as evidenced by the low standard deviation values, implies that the tool’s accuracy metric is well-received among users across the two groups. These results highlight the potential utility of the benchmark framework and the IoT traffic analyzer tool in measuring and analyzing the network behavior of IoT devices in smart home environments.

## 5. Conclusions and Future Work

This research differs from other IoT researches as it aims to assist the Internet of Things research community in facilitating their research so that they focus on the research contribution. Accordingly, we examined all previous IoT research that focused on analyzing the network traffic in order to extract their features for various purposes. We highlighted a fundamental difference between the network traffic character of IoT devices and non-IoT devices such as smartphones or PCs. Also, we proved that the process of analyzing IoT traffic is essential to pursue the research regardless of its contribution.

Accordingly, we built a public IoT analyzer tool that can automatically analyze any IoT device’s network traffic comprehensively and reliably at the packet and flow level. The data were collected from four different IoT devices. For each device, we applied different scenarios; In total, seventeen (17) scenarios were implemented. As a result, the tool successfully extracted all possible features and classified them into three CSV files, each of which contains different characteristics. For example, the first CSV file has 24 features from analyzing each packet individually between the IoT device and the server/s or between the IoT device and other IoT devices. In contrast, the second CSV file has 16 features from analyzing the communication pattern of a particular IoT device. Finally, the third CSV file contains 15 statistical features calculated from a high-level overview of network statistics regarding the behavior of the IoT device within the network. Such extracted features can help the IoT researcher to identify either:

1. The type of IoT device type, i.e., smart light, smart camera, etc. or
2. The function of the IoT device, i.e., whether the device is on, off, or idle, or
3. Whether there is any human interaction with the IoT device and what this interaction is, or
4. The behavior of the IoT within the network, i.e., if the IoT interacts with other IoT or non-IoT devices that are connected to the same network, or
5. Whether the IoT device is under attack and what type of this attack, i.e., MITM attack or IP address spoofing detection and so on.

Consequently, through these files, the researcher can pick or specify the features that suit his research; then create a database for the behavior of each IoT device.

To test the tool’s performance and effectiveness in analyzing IoT traffic and producing correct results, we experimented it on several IoT researchers at different stages of their research and classify them into three groups. The first group of researchers who are finished their research, the second group of researchers is in the middle of their research, and finally, the third group of researchers is at the beginning of their research. Based on their feedback, the tool has proven its reliability, trustworthiness, and comprehensiveness in analyzing the IoT traffic and extracting all the possible features. In addition, they were very satisfied with the results of the three generated CSV files, which helped them to pursue their research smoothly. Also, they found that the description of each feature, as well as the importance and how such features will be used, are beneficial as they can use it as a guide for which feature/s they can select to suit their research. On the other hand, in terms of speeding up the search, the researchers clarified that the tool effectively shortens the time needed to write code for analyzing the IoT traffic and extracting useful features.

Future work that extends on our findings might address the incorporation of automated machine learning techniques which classify the numerical input of the IoT network behavior. In addition, we will conduct future enhancements of this tool to generate well-constructed datasets for each IoT device to ensure the scalability of such a tool.

## Figures and Tables

**Table 2:** IoT devices used for smart home.

No.	IoT Device	Brand	Description
1	Echo Dot (4th generation). Smart speaker with Alexa (Arabic or English)	Amazon	It can play songs and connect to external speakers or headphones. It is capable of making calls and messaging with voice commands. It is capable of hearing your voice from all directions, even when songs are played. Controls compatible with smart home devices, including lights, plugs, and more.
2	Tapo C200 Pan/Tilt 1080p Full HD Home Security Wi-Fi Camera	TP-link	Wireless Indoor Security 360° 2Mp 1080P (Full Hd). Works with Alexa and Google (Tapo C200)
3	TP-LINK Tapo Smart Plug Wi-Fi Outlet,	TP-link	Instantly turn connected devices on/off wherever you are through the Tapo app and reset a schedule to manage devices automatically. Create countdown timer lists for connected electronics. Manage your smart plug with voice commands via Amazon Alexa or Google Assistant. Automatically turns devices on and off at different times to give the appearance that someone is home
4	TP-Link Kasa Smart LED Lamp Multi-Color	TP-link	Multicolor with a wide range of colors and dimming capabilities, Kasa smart’s multicolor light bulb offers endless lighting possibilities. No hub required; connects to your home’s secure Wi-Fi network

**Table 3:** Different scenarios of collecting the IoT traffic except for Alexa.

IoT Device	Scenarios/ Experiments	Power is on (True/False)	App is on (True/False)	Device Idle (True/False)	App Idle (True/False)	User Interaction with IoT App (True/False)
Smart Plug	Scenario 1	True	True	True	True	False
	Scenario 2	Alternating	True	False	False	True
	Scenario 3	True	False	True	Na	Na
	Scenario 4	False	True	True	True	False
	Scenario 5	False	False	True	Na	Na
Smart lamb	Scenario 1	True	True	True	True	False
	Scenario 2	Alternating	True	False	False	True
	Scenario 3	True	False	True	Na	False
	Scenario 4	False	True	True	True	False
	Scenario 5	False	False	True	Na	Na
Smart Cam	Scenario 1	True	True	Na	Na	Na
	Scenario 2	False	False	Na	Na	Na

**Table 4:** Different scenarios of collecting the traffic from Alexa.

IoT Device	Scenarios/ Experiments	App Idle (True/False)	Play an Audio (True/False)	Connect to a Website (True/False)	Connect to Other IoT Devices (True/False)
Alexa	Scenario 1	True	False	False	False
	Scenario 2	True	False	True	False
	Scenario 3	False	True	False	False
	Scenario 4	True	True	False	False
	Scenario 5	False	False	False	True

**Figure A1:**
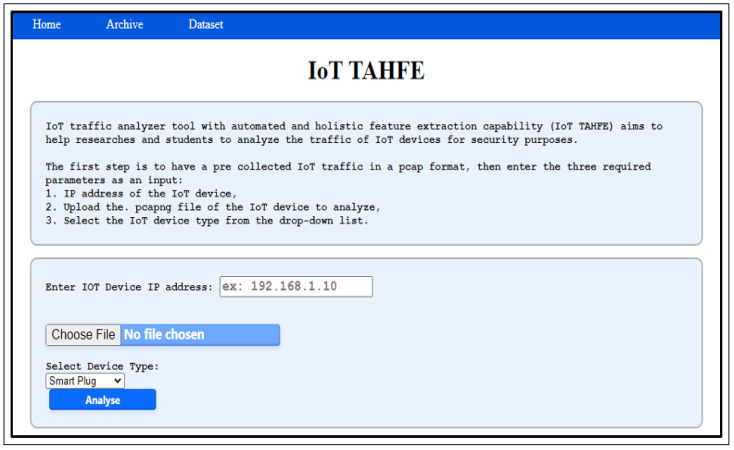
The first Stage in the TAHFE tool-Homepage.

**Figure A2:**
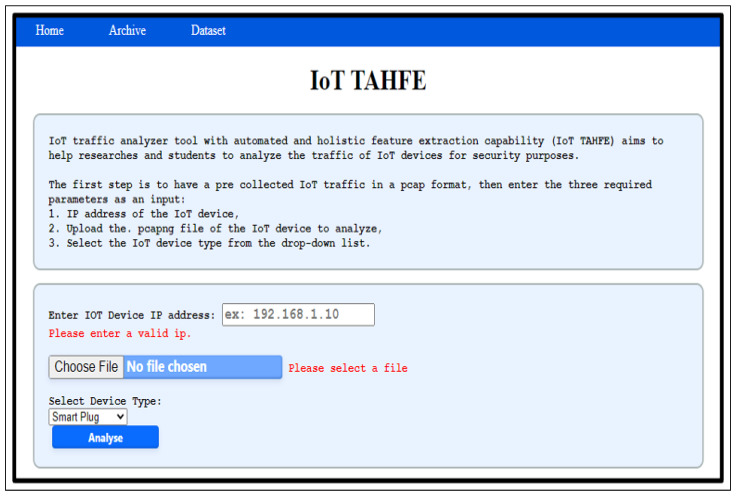
The checking on the validation of the IP.

**Figure A3:**
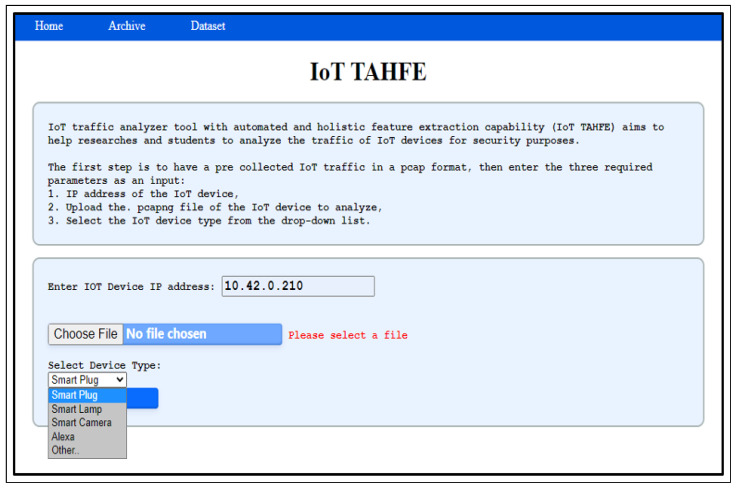
The checking on the validation of selected file.

**Figure A4:**
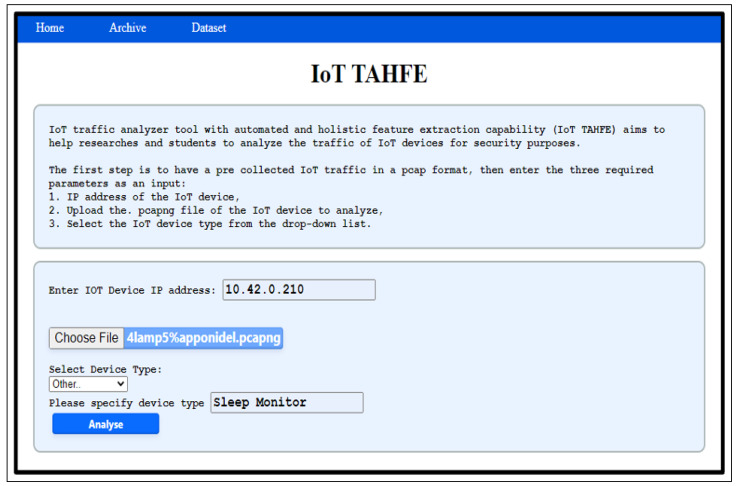
The screen where all required inputs are correct.

**Figure A5:**
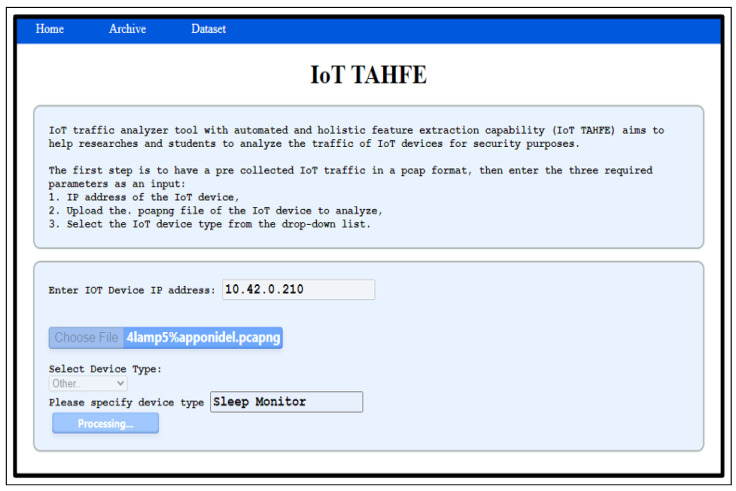
The screen of starting the analyzing process and checking whether the IoT IP address is correct or not.

**Figure A6:**
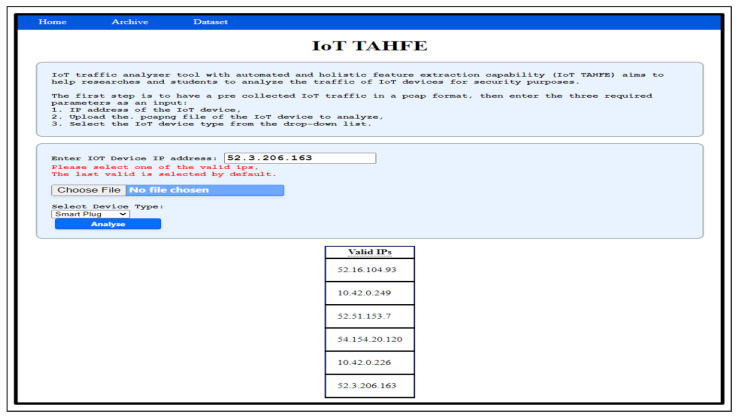
The screen shows that the entered IoT IP address is wrong. Display all valid IP’s in the file.

**Figure A7:**
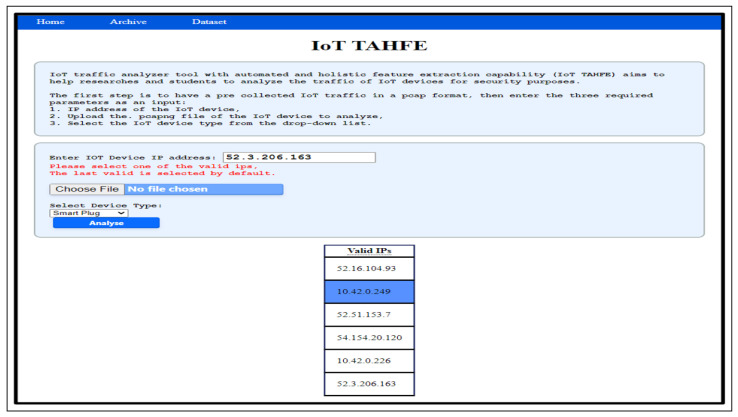
The screen shows the user select one of valid IP.

**Figure A8:**
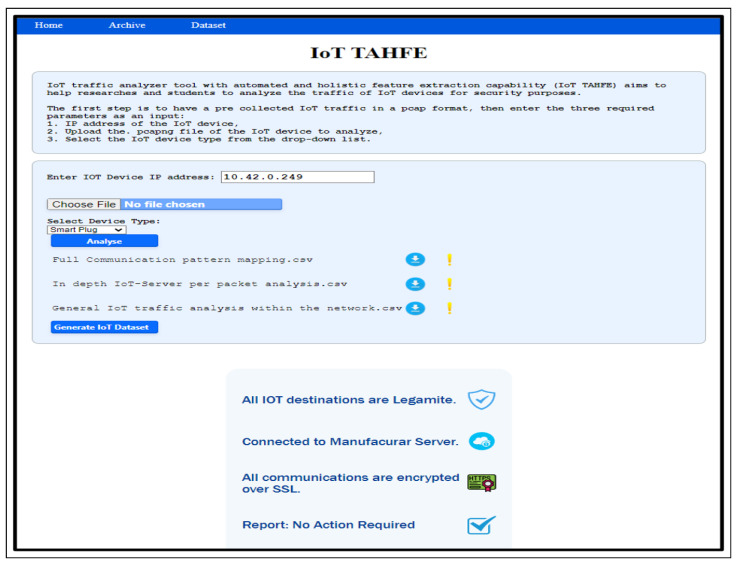
The screen of starting the analyzing process.

**Figure A9:**
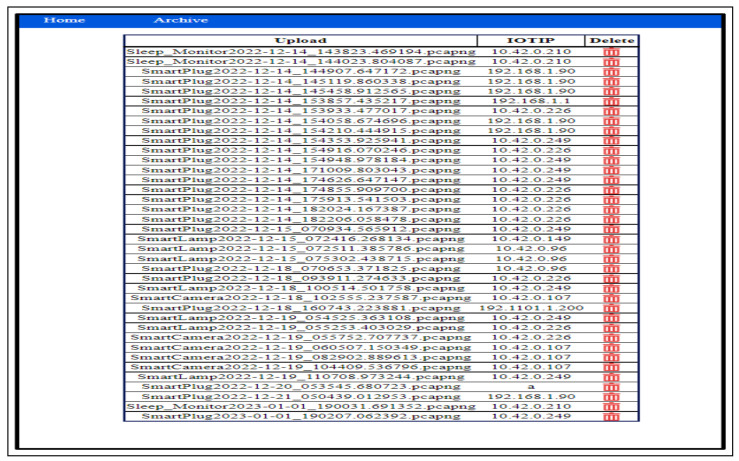
The screen of archived files.

## Data Availability

The tool is available to all IoT researchers through the following link: http://iottrafficanalyzer.com/, (accessed on 15 May 2023).The source code of the tool is available on the GitHub page at the following link: https://github.com/Malmasre/TAHFE_IOTtrafficAnalyzer, (accessed on 15 May 2023).

## Abbreviations

The following abbreviations are used in this manuscript:

IoT	Internet of Things
IoT TAHEF	IoT Traffic Analyzer Tool with Automated and Holistic Feature Extraction Capability

## Appendix A. The Manual of IoT ATAFED Tool

To access the IoT ATAFED tool, open a browser and write the following URL: (http://iottrafficanalyzer.com/) (accessed on 15 May 2023), then follow the following steps:

The first stage is to provide the tool with the required inputs, Figure A1, Figure A2 and Figure A3 illustrate the initial screen where the user can input three main values:

- IP address of the IoT device.
- Upload the. pcapng file of the IoT device to analyze.
- Select the IoT device type from the drop-down list.

Notice that if the device type is not in the list, the user can choose another and write the device type. Also, the user can analyze one pcap file at a time.

The second stage is to check the validity of the inputs. In this step, the tool will run several checkups:

- It will check whether the IP address is typed correctly; otherwise, an error message appears to inform the user to enter the correct IP format.
- the tool will check whether the uploaded file is in pcap file format or not. Otherwise, it will give an error message to upload the correct file format, as shown in Figure A4.

In the third stage, the user is ready to analyze the pcap file by clicking on the “Analyze” button, as shown in Figure A5.

However, during the analysis time, another essential checkup happens. The tool ensures that the IoT IP address entered by the user is included in the pcap file.

Otherwise, the tool returns a clickable list of valid IPs which are not server IP addresses. Then, the tool will select the last valid IP from the IP list by default; however, the user can edit the IP address by selecting the required one from the list or typing it manually, as shown in Figure A5, Figure A6 and Figure A7.

Once the user types the correct IP address, the file needs to be uploaded again, then click on the “Analyze” button to start the analysis process,

In the last stage, As shown in Figure A8, the tool analyzes the pcap file in-depth according to the selected IoT IP address. As a result, three different CSV files are generated, each of which has different features as well as different network properties. The user can download the files by clicking on the download icon in front of each file.

Finally, the entire analysis history can be viewed, downloaded and deleted from the archive tap, Figure A9.

## Appendix B. The Evaluation Survey Form

The following form to help us evaluate the tool and check its performance.

The General Questions:

What is your IoT research academic level

- IT Researcher
- Ph.D. researcher
- Master researcher
- Bachelor researcher

What is the closest topic for your IoT research topic:

- Machine Learning with IoT
- IoT traffic analyzing
- Profiling IoT device
- Intrusion detection systems
- Identify the type and model of IoT device
- IoT behavior fingerprint
- Other:

What is the stage of you research

- IoT research is completed
- IoT research is still in the middle
- IoT research is not started yet

**Table A1:** Criteria metrics to evaluate the effectiveness and the efficiency of the IoT TAHEF tool.

	Strongly Agree	Agree	Neutral	Strongly Disagree	Disagree
The interface of the tool is user friendly					
The tool has clear fields for input.					
The tool provides assistance on invalid input.					
The tool is fault tolerant. (The tool can handle a wrong input).					
The tool has clear process/downloads buttons.					
The tool generates the files in a reasonable time (less than 2 min)					
The tool automatically displays the results upon finishing the processing.					

**Table A2:** The extracted features help the IoT researcher to.

	Strongly Agree	Agree	Neutral	Strongly Disagree	Disagree
Recognize the IoT device type, i.e., smart light, smart camera					
Identify the IoT device behavior, i.e., whether the device is on, of, or idle.					
Determine human interaction type.					
Identify the behavior of the IoT device within the network.					
Recognize abnormal behavior, i.e., attacks such as MITM detection or IP address spoofing detection.					

Does the IoT TAHEF tool help in speed up the research time

- Strongly agree
- Agree
- I don’t Know
- Disagree
- Strongly disagree

Does the IoT TAHEF tool help in creating a dataset for the IoT device based on the extracted features?

- Strongly agree
- Agree
- I don’t Know
- Disagree
- Strongly disagree

Does the IoT TAHEF tool help in aiding IoT researchers regardless of their contribution/s in analyzing the IoT traffic for security purposes?

- Strongly agree
- Agree
- I don’t Know
- Disagree
- Strongly disagree
